# All-cause mortality effects of replacing sedentary time with physical activity and sleeping using an isotemporal substitution model: a prospective study of 201,129 mid-aged and older adults

**DOI:** 10.1186/s12966-015-0280-7

**Published:** 2015-09-30

**Authors:** Emmanuel Stamatakis, Kris Rogers, Ding Ding, David Berrigan, Josephine Chau, Mark Hamer, Adrian Bauman

**Affiliations:** Charles Perkins Centre, University of Sydney, Sydney, Australia; Exercise and Sport Sciences, Faculty of Health Sciences, University of Sydney, Sydney, Australia; UCL-PARG (Physical Activity Research Group), Department of Epidemiology and Public Health, University College London, London, UK; Prevention Research Collaboration, Sydney School of Public Health, University of Sydney, Sydney, Australia; Graduate School of Public Health, San Diego State University, San Diego, CA USA; National Cancer Institute, Behavioral Research Program, Division of Cancer Control and Population Sciences, Bethesda, MD USA; National Centre for Sport & Exercise Medicine, Loughborough University, Loughborough, UK

**Keywords:** Physical activity, Sedentary behaviour, Mortality, Longitudinal, Isotemporal substitution, Prevention, Sitting, Screen time, Mortality, Epidemiology, Sleeping, Public health, Population cohort

## Abstract

**Background:**

Sedentary behaviour, sleeping, and physical activity are thought to be independently associated with health outcomes but it is unclear whether these associations are due to the direct physiological effects of each behaviour or because, across a finite 24-hour day, engagement in one behavior requires displacement of another. The aim of this study was to examine the replacement effects of sedentary behaviour (total sitting, television/computer screen time combined), sleeping, standing, walking, and moderate-to-vigorous physical activity on all-cause mortality using isotemporal substitution modelling.

**Methods:**

Longitudinal analysis (4.22 ± 0 · 9 years follow-up/849,369 person-years) of 201,129 participants of the 45 and Up study aged ≥45 years from New South Wales, Australia.

**Results:**

Seven thousand four hundred and sixty deaths occurred over follow-up. There were beneficial associations for replacing total sitting time with standing (per-hour HR: 95 % CI: 0.95, 0.94–0.96), walking (0.86, 0.81–0.90), moderate-to-vigorous physical activity (0.88, 0.85–0.90), and sleeping in those sleeping ≤ 7 h/day (0.94, 0.90–0.98). Similar associations were noted for replacing screen time. Replacing one hour of walking or moderate-to-vigorous physical activity with any other activity class was associated with an increased mortality risk by 7–18 %. Excluding deaths in the first 24 months of the follow up and restricting analyses to those who were healthy at baseline did not materially change the above observations.

**Conclusion:**

Although replacing sedentary behaviour with walking and moderate-to-vigorous physical activity are associated with the lowest mortality risk, replacements with equal amounts of standing and sleeping (in low sleepers only) are also linked to substantial mortality risk reductions.

**Electronic supplementary material:**

The online version of this article (doi:10.1186/s12966-015-0280-7) contains supplementary material, which is available to authorized users.

## Background

The health benefits of moderate-to-vigorous physical activity (MVPA) are well-established [1]. Large volumes of sedentary behaviour (SB), characterized by a low energy expenditure (≤1.5 metabolic equivalents) in a sitting or reclining posture [2], are thought to increase mortality risk independently of MVPA [3, 4]. The evidence is particularly strong for specific types of SB, such as television viewing [5, 6], which show consistent associations with health outcomes. The associations between sleep duration and health outcomes are complex, as both low and high durations are associated with mortality risk [7].

Although physical activity (PA), SB, and sleeping are behaviours that occupy a 24-hour day, investigators have typically examined each behaviour without considering what time-dependent behaviours are being displaced [4, 7–9]. For example, a 60-minute block of low-intensity walking will have different health effects depending on whether it displaces an equal amount of sitting, vigorous exercise, or brisk walking. This limitation can be overcome by statistical modeling that specifically estimates the effects of replacing one behaviour with another, the isotemporal substitution modeling [ISM] approach [10]. ISM, which is based on nutritional epidemiology methods analyzing the effects of substituting nutritional components [10], models simultaneously the effect of a given activity being performed and another activity being displaced in an equal time-exchange manner. ISM not only controls for confounding by other time-dependent behaviours, it also captures the effect of time substitution [10]. Very few studies to date have examined the replacement associations of SB, physical activity and other time-dependent behaviours with health outcomes in general [10–12] and no such study has had mortality as an outcome. The only study that has specifically considered the replacement associations of MVPA, SB, and sleeping was a cross-sectional study that found beneficial associations of replacing sedentary time with sleeping and MVPA and a number of cardiometabolic risk markers [12]. However, “sedentary time” in this study [12] did not distinguish between sitting and standing. Another recent cross-sectional ISM study [11] found that replacing sedentary time with MVPA, but not with light-intensity activity, was linked to beneficial associations with a range of cardiometabolic markers. Such information has both clinical and public health relevance and may be valuable in developing more accurate and specific public health recommendations, clinical guidelines, and preventative or therapeutic interventions.

In this study, we examined the estimated replacement effects of SB and other time-dependent behaviours on all-cause mortality in a large population-based cohort of Australians aged 45 years and over using isotemporal substitution modelling.

## Methods

### Sample

The analyses are based on data from the 45 and Up Study [4, 13], in which participants completed a baseline questionnaire from January 2006 through December 2009. The 45 and Up Study sample is a large-scale (*N* = 267,119) prospective cohort of men and women aged 45 years or older living in the state of New South Wales, Australia. Participants were randomly sampled from the Medicare Australia (the national universal health care) database, which primarily includes all citizens and permanent residents of Australia. Eligible individuals were mailed all study materials and were asked to complete and mail the questionnaire and consent forms to the study center. The overall 45 & Up response rate to the mailed invitations was 17 · 9 % (95 % CI 17 · 8–18 · 1) and the final sample size corresponded to 11 % of the New South Wales population of the target age group [13]. The present project was approved by the New South Wales Population and Health Services Research Ethics Committee (reference No. 2010/05/234). Additional file 1: Figure S1 describes the selection of the analytic sample. From the 267,119 respondents, we excluded those with a missing or invalid date of recruitment (missing *n* = 12, implausible date *n* = 2184) and the resulting 265,923 participants were linked to the New South Wales Registry of Births, Deaths, and Marriages (RBDM) database. We then excluded one RBDM death record that had no matching record and any linked records where the date of death occurred before recruitment (*n* = 20), to form an initial dataset (*n* = 264,903). During the data cleaning process, 63,774 participants with or implausible exposure or covariate values were excluded, leaving a core analytic sample of 201,129 participants.

### Exposure variables

All study variables were assessed through a self-administered questionnaire. Information on the main exposures was based on self-reported data from the 45 and Up Study baseline questionnaire (available at https://www.saxinstitute.org.au/our-work/45-up-study/questionnaires/). The sitting, screen time (watching television or using a computer), standing and sleeping variables were assessed with the question “About how many hours in each 24-hour day do you usually spend doing the following?” followed by open entry boxes where participants entered their responses. This type of question is similar to the validated sitting questions of the International Physical Activity Questionnaire [14]. Total weekly time for walking and non-walking MVPA (continuously for at least 10 min) was assessed using the Active Australia Survey questions: “If you add up all the time you spent doing each activity last week, how much time did you spend altogether doing each type of activity?” These questions have been shown to have acceptable reliability (coefficients for walking, moderate and vigorous activity frequency, and time ranging from 0.56 to 0.64) and validity (correlation of duration of self-reported activity with accelerometer data was 0.52) [15, 16] and have been tested both in population and individual-level intervention contexts [17].

### Potential confounders

The choice of potential confounders was similar to previous 45 & Up SB analyses [4] and included sex, age (5-year bands), educational level (university degree/post high school, high school, or less), marital status (single/married/cohabiting/widowed/divorced/separated), urban/rural residence, body mass index (calculated as self-reported weight/self-reported height squared [18]), smoking status (current/previous/never smoker), self-rated health (poor/fair/good/very good/excellent), help with daily tasks because of long-term illness or disability, psychological distress (K10 scale, a 10-item questionnaire intended to yield a global measure of anxiety and depressive symptoms [19]), and previous physician diagnoses of CVD, diabetes mellitus, or various types of cancer.

### Outcome ascertainment

All-cause mortality was ascertained from the New South Wales RBDM from February 1, 2006 through June 14, 2012. Mortality data were linked to the baseline data from the 45 and Up Study by the Centre for Health Record Linkage (New South Wales, Australia) using linkage methods and quality checks that have been described previously [4].

### Data handling

The Supplementary Methods in Additional file 1 presents the unabridged version of data cleaning, handling, and statistical testing procedures. In summary, we used multiple imputation (SAS 9.3, Proc MI) and the Expectation-Maximisation algorithm (30 imputations) [20] to impute missing data for the 13,053 participants who had at least one of the time-dependant behavioural variables. Due to the ISM requirement for an approximately linear association between each exposure and the outcome, moderate and vigorous PA were combined into MVPA. For the same reason [7, 9] we treated sleeping as a piecewise variable with a breakpoint at 7 h (≤7 h/day and > 7 h/day), where each of the two sleeping variables had an approximately linear association with mortality. All exposure variables were converted into hours per day.

### Statistical analyses

The association between each activity class and risk of death was analyzed using Cox proportional hazards regression models. Survival time (in weeks) was measured as the time from baseline to death or the censor point, and each exposure was modelled in one-hour intervals. Before ISM analyses, all relevant assumptions were tested. Interactions of sex and age were not statistically significant (all *p* > 0 · 10); therefore results are presented for the entire sample. We initially estimated the partition model, which assumes that each activity class is added rather than substituted with another activity to create a day potentially longer than 24 h [10]. The partition model estimates each component of time while keeping others constant (sleep_a and sleep_b correspond to daily sleep durations of ≤ 7 h and > 7 h, respectively):

$$ \begin{array}{l} \log {h}_i(t)={b}_0(t)+{b}_1\left(\mathrm{sleep}\_\mathrm{a}\right)+{b}_2\left(\mathrm{sleep}\_\mathrm{b}\right)+{b}_3\left(\mathrm{sitting}\right)+{b}_4\left(\mathrm{standing}\right)\\ {}+{b}_5\left(\mathrm{walking}\right)+{b}_6\left(\mathrm{MVPA}\right)+{b}_7\left(\mathrm{screntime}\right)+\left(\mathrm{covariates}\right)\end{array} $$

ISM assumes that any given time spent in one behaviour will lead to an isotemporal displacement of another activity class while total time is kept constant [10]. For example, to estimate the effect of substituting one hour of standing for screen-time, screen-time is removed from a model adjusted for total time as follows:

$$ \begin{array}{l} \log {h}_i(t)={b}_0(t)+{b}_1\left(\mathrm{sleep}\_\mathrm{a}\right)+{b}_2\left(\mathrm{sleep}\_\mathrm{b}\right)+{b}_3\left(\mathrm{sitting}\right)+{b}_4\left(\mathrm{standing}\right)\\ {}+{b}_5\left(\mathrm{walking}\right)+{b}_6\left(\mathrm{MVPA}\right)+{b}_7\left(\mathrm{Total}\right)+\left(\mathrm{covariates}\right)\end{array} $$

In the above example, the resulting hazard ratio (HR) for standing will indicate whether replacing screen time with standing is beneficially (if HR < 1 · 00) or detrimentally (if HR > 1 · 00) associated with all-cause mortality. We repeated a series of sensitivity analyses to examined the robustness of our results: a) using the unimputed dataset only; b) excluding those with pre-existing CVD (heart disease, stroke, and thrombosis), or diabetes, or cancer at baseline; c) excluding deaths in the first 24 months (*n* = 4714); d) excluding both b and c above, e) alternative manipulations of the SB variables are performed and shown in the Supplementary Appendix; f) stratifying by sleep time; g) change the cut-off of the piecewise sleep variable to 8 h/day. For comparability with a recent study that examined the associations between standing and all-cause mortality, we repeated the partition models of the standing time exposure stratified by MVPA level using the 150 MVPA minutes/week as the cut off point for adherence to the World Health Organisation physical activity recommendations. Analyses were performed using SAS Software version 9 · 3 (SAS Institute Inc., Cary, NC). The reporting of this study conforms to the STROBE statement.

## Results

During a mean-follow up time of 4.22 years (849,369 total person-years), 7,460 deaths were recorded (3 · 7 % of the sample). Table 1 presents participant characteristics by daily sitting time. Additional file 1: Table S1 presents the descriptive statistics of all exposure variables. Table 2 presents the partition and ISM results for the full imputed dataset. There were beneficial associations for replacing sitting with sleeping in those sleeping for ≤7 h/day (HR per hour increase: 0.94, 95 % CI: 0.90–0.98), with standing (0.95, 0.94–0.96), with walking (0.86, 0.81–0.90), and with MVPA (0.88, 0.85–0.90); and for replacing screen time with sleeping in those sleeping for ≤ 7 h/day, (0.95, 0.91 to 0.99), standing (0.97, 0.95–0.98), walking (0.87, 0.82–0.92) and MVPA (0.89, 0.86–0.91). Sleeping for >7 h (1.08, 1.05 -1.10), screen time (1.02, 1.01- 1.03), and sitting (1.03, 1.02–1.04) were associated with increased mortality risk. Standing (0.97, 0.96–0.99), walking (0.83, 0.79–0.88), and MVPA 0.87 (0.85–0.90) were all associated with decreased risk. Among those who reported sleeping >7 h/day, replacing one hour of sleeping with one hour of any other activity class was associated with risk reductions (Table 2). Replacing walking or MVPA with any other behaviour was associated with increased risk. All these replacement effects are also presented graphically in Additional file 1: Figure S2. All above observations were not materially different when we repeated analyses to the unimputed dataset (Additional file 1: Table S3). When analyses were restricted to healthy at baseline only participants (*n* = 143,680; 2690 deaths) associations in the partition models were somewhat attenuated, although the ISM results for screen time, sitting, standing, walking and MVPA followed broadly the same pattern as in the full sample (Table 3) and the same applied to the remaining 57,449 participants (4770 deaths) who had diabetes, or CVD or a history of cancer at baseline (Additional file 1: Table S4). Excluding those 4714 participants who died during the 24 months of follow up did not materially affect the results (Table 4). Restricting analyses to those who were healthy at baseline and excluding events occurring the first 24 months (*n* = 142,768; 1,778 deaths) resulted in broadly similar results, although associations were generally attenuated and confidence intervals were broader due to the dilution of the events rate (Additional file 1: Table S5). Different manipulations of the sitting and screen time variables (Additional file 1: Table S6-S7) did not materially change the above results. Neither stratifying analyses by sleeping time level nor changing the piecewise sleeping variable cutoff to 8 h/day changed results materially (Additional file 1: Tables S8-S10). The standing time partition models stratified by MVPA suggested that the beneficial effect of standing on mortality was present in both those who met (per-hour HR: 0.98, 0.97–0 · 99) and did not meet (0.95, 0.94–0.97) the World Health Organization physical activity recommendations.

**Table 1:** Characteristics (*n*, % (of sitting time group)) of participants by amount of sitting time per day

		0–2 hrs	3–4 hrs	5–6 hrs	7+ hrs
Gender	Men	16,516 (43.9 %)	27,993 (46.3 %)	24,719 (47.5 %)	24,713 (51.9 %)
	Women	21,116 (56.1 %)	32,477 (53.7 %)	27,316 (52.5 %)	22,908 (48.1 %)
Age at baseline	45–49 yrs	6,249 (16.6 %)	7,764 (12.8 %)	6,405 (12.3 %)	8,257 (17.3 %)
	50–54 yrs	7,104 (18.9 %)	9,991 (16.5 %)	7,979 (15.3 %)	10,093 (21.2 %)
	55–59 yrs	7,373 (19.6 %)	10,433 (17.3 %)	8,933 (17.2 %)	9,280 (19.5 %)
	60–64 yrs	6,231 (16.6 %)	9,850 (16.3 %)	8,191 (15.7 %)	6,401 (13.4 %)
	65–69 yrs	4,470 (11.9 %)	8,300 (13.7 %)	7,140 (13.7 %)	4,355 (9.1 %)
	70–74 yrs	2,759 (7.3 %)	5,755 (9.5 %)	5,018 (9.6 %)	2,898 (6.1 %)
	75–79 yrs	1,645 (4.4 %)	3,773 (6.2 %)	3,615 (6.9 %)	2,291 (4.8 %)
	80–84 yrs	1,380 (3.7 %)	3,443 (5.7 %)	3,483 (6.7 %)	2,645 (5.6 %)
	85+ yrs	421 (1.1 %)	1,161 (1.9 %)	1,271 (2.4 %)	1,401 (2.9 %)
Education level	University degree	7,731 (20.8 %)	12,952 (21.7 %)	12,615 (24.5 %)	15,760 (33.4 %)
Marital status	Married/in relationship	29,573 (79.0 %)	46,961 (78.0 %)	39,804 (76.9 %)	35,563 (75.1 %)
Region type	Major city	15,177 (40.3 %)	25,327 (41.9 %)	23,365 (44.9 %)	25,062 (52.6 %)
	Inner regional	13,932 (37.0 %)	22,177 (36.7 %)	18,847 (36.2 %)	15,268 (32.1 %)
	Outer regional/remote	8,523 (22.6 %)	12,966 (21.4 %)	9,823 (18.9 %)	7,291 (15.3 %)
Body mass index	Obese (≥30 Kg/m^2^)	7,203 (20.6 %)	11,839 (21.0 %)	10,995 (22.6 %)	11,326 (25.3 %)
Smoking status	Current smoker	2,914 (7.8 %)	4,431 (7.3 %)	3,592 (6.9 %)	3,402 (7.2 %)
Self-rated health	Excellent	6,568 (18.0 %)	9,260 (15.8 %)	7,296 (14.4 %)	6,712 (14.5 %)
	Very good	14,370 (39.3 %)	22,924 (39.0 %)	19,250 (38.0 %)	16,808 (36.2 %)
	Good	11,714 (32.1 %)	19,707 (33.5 %)	17,306 (34.2 %)	15,653 (33.7 %)
	Fair	3,366 (9.2 %)	6,039 (10.3 %)	5,909 (11.7 %)	6,070 (13.1 %)
	Poor	515 (1.4 %)	810 (1.4 %)	882 (1.7 %)	1,181 (2.5 %)
Require help for disability		1,216 (3.4 %)	2,176 (3.8 %)	2,401 (4.8 %)	2,814 (6.1 %)
Psychological distress	High distress	4,570 (7.7 %)	3,463 (7.5 %)	2,304 (7.0 %)	3,057 (6.9 %)
History of CVD		11,458 (18.0 %)	8,954 (17.7 %)	5,988 (16.7 %)	6,656 (13.8 %)
History of cancer		5,683 (8.9 %)	4,554 (9.0 %)	3,051 (8.5 %)	3,746 (7.8 %)
History of diabetes		5,777 (9.1 %)	4,418 (8.8 %)	2,836 (7.9 %)	3,060 (6.3 %)
Died		3,083 (4.7 %)	2,066 (4.0 %)	1,196 (3.3 %)	1,115 (2.3 %)

**Table 2:** Independent^a^ and replacement^b^ effects of sleeping, screen time, sitting, walking and non-walking moderate to vigorous physical activity on all-cause mortality risk. Imputed data^c^ (*n* = 201,129; 7,460 deaths)

	With 1 hr of:							
1. Isotemporal Substitution Model^b^ *Replace 1 hr of*:	Sleeping (≤7 hrs)	Sleeping (>7 hrs)	Screen-time	Sitting	Standing	Walking	MVPA	Total activity
A. Sleeping (≤7 hrs)	-	-	1.01 (0.98–1.05)	1.03 (0.99–1.07)	0.98 (0.94–1.02)	0.93 (0.84–1.03)	0.90 (0.85–0.96)	1.01 (0.98–1.04)
B. Sleeping (>7 hrs)	-	-	0.95 (0.93–0.97)	0.96 (0.94–0.98)	0.92 (0.9–0.94)	0.80 (0.75–0.86)	0.84 (0.81–0.87)	1.06 (1.04–1.07)
C. Screen-time	0.95 (0.91–0.99)	1.06 (1.04–1.09)	-	1.01 (1.00–1.03)	0.97 (0.95–0.98)	0.87 (0.82–0.92)	0.89 (0.86–0.91)	1.01 (1.00–1.02)
D. Sitting	0.94 (0.90–0.98)	1.05 (1.03–1.07)	0.99 (0.97–1.00)	-	0.95 (0.94–0.96)	0.86 (0.81–0.90)	0.88 (0.85–0.90)	1.02 (1.01–1.03)
E. Standing	0.99 (0.95–1.03)	1.10 (1.08–1.13)	1.04 (1.02–1.05)	1.05 (1.04–1.06)		0.90 (0.85–0.95)	0.92 (0.89–0.95)	0.98 (0.97–0.99)
F. Walking	1.10 (1.03–1.18)	1.17 (1.12–1.21)	1.15 (1.09–1.22)	1.17 (1.11–1.23)	1.11 (1.05–1.18)	-	1.02 (0.96–1.09)	0.88 (0.83–0.93)
G. MVPA	1.07 (1.02–1.13)	1.18 (1.14–1.22)	1.13 (1.09–1.16)	1.14 (1.11–1.18)	1.09 (1.06–1.12)	0.98 (0.92–1.04)	-	0.90 (0.87–0.92)
2. Partition model^a^	1.01 (0.98–1.04)	1.08 (1.06–1.10)	1.02 (1.01–1.03)	1.03 (1.02–1.04)	0.97 (0.96–0.99)	0.83 (0.79–0.88)	0.87 (0.85–0.90)	

^a^Adjusted for sex, age, educational level, marital status, urban or rural residence, BMI, smoking status, self-rated health, receiving help with daily tasks for a long-term illness or disability, prevalent disease at baseline (cardiovascular disease, diabetes, or cancer), psychological distress, and mutually adjusted for all activity classes

^b^Adjusted for sex, age, educational level, marital status, urban or rural residence, BMI, smoking status, self-rated health, receiving help with daily tasks for a long-term illness or disability, (cardiovascular disease, diabetes, or cancer), psychological distress, mutually adjusted for all activity classes, and total time in all activity classes

^c^Multiple imputation to replace missing time of the activity classes (based on age, sex, and non-missing other activity classes variables)

**Table 3:** Independent^a^ and isotemporal substitution^b^ effects of sleeping, screen time, sitting, walking and non-walking moderate to vigorous physical activity on all-cause mortality risk. Participants who were considered healthy at baseline, defined as those who were never diagnosed with cardiovascular disease, diabetes, or cancer, (Imputed data^c^, *n* = 143,680; 2690 deaths)

	With 1 hr of:							
1. Isotemporal Substitution Model- *Replace 1 hr of*:	Sleeping (<=7 hrs)	Sleeping (>7 hrs)	Screen-time	Sitting	Standing	Walking	MVPA	Total activity
A. Sleeping (<=7 hrs)	-	-	1.03 (0.97–1.09)	1.05 (0.98–1.11)	1.02 (0.96–1.08)	0.95 (0.81–1.11)	0.95 (0.86–1.04)	0.99 (0.94–1.05)
B. Sleeping (>7 hrs)	-		0.98 (0.94–1.01)	0.98 (0.95–1.02)	0.94 (0.91–0.98)	0.88 (0.79–0.98)	0.89 (0.84–0.95)	1.04 (1.00–1.07)
C. Screen-time	0.89 (0.83–0.95)	1.06 (1.02–1.1)	-	1.01 (0.99–1.04)	0.97 (0.95–0.99)	0.91 (0.84–0.99)	0.92 (0.87–0.96)	1.01 (1.00–1.03)
D. Sitting	0.88 (0.82–0.94)	1.04 (1.00–1.08)	0.99 (0.96–1.01)	-	0.96 (0.94–0.98)	0.90 (0.83–0.98)	0.90 (0.86–0.95)	1.03 (1.01–1.04)
E. Standing	0.91 (0.85–0.97)	1.08 (1.04–1.12)	1.03 (1.01–1.05)	1.04 (1.02–1.06)	-	0.93 (0.86–1.02)	0.94 (0.90–0.98)	0.99 (0.97–1.00)
F. Walking	0.98 (0.88–1.08)	1.18 (1.11–1.26)	1.10 (1.01–1.20)	1.11 (1.02–1.21)	1.07 (0.98–1.16)	-	1.01 (0.91–1.11)	0.92 (0.85–1.00)
G. MVPA	0.97 (0.90–1.05)	1.17 (1.11–1.23)	1.09 (1.04–1.14)	1.11 (1.06–1.16)	1.06 (1.02–1.11)	0.99 (0.90–1.10)	-	0.93 (0.89–0.97)
2. Partition model^a^	0.99 (0.94–1.05)	1.06 (1.03–1.10)	1.02 (1.01–1.04)	1.03 (1.02–1.05)	0.98 (0.97–0.99)	0.89 (0.82–0.97)	0.91 (0.87–0.95)	-

^a^Adjusted for sex, age, educational level, marital status, urban or rural residence, BMI, smoking status, self-rated health, receiving help with daily tasks for a long-term illness or disability, prevalent disease at baseline (cardiovascular disease, diabetes, or cancer ), psychological distress, and  mutually adjusted for all activity classes

^b^Adjusted for sex, age, educational level, marital status, urban or rural residence, BMI, smoking status, self-rated health, receiving help with daily tasks for a long-term illness or disability, psychological distress, mutually adjusted for all activity classes, and total time in all activity classes

^c^Multiple imputation to replace missing time of the activity classes (based on age, sex, and non-missing other activity classes variables)

**Table 4:** Independent^a^ and isotemporal substitution^b^ effects of sleeping, screen time, sitting, walking and non-walking moderate to vigorous physical activity on all-cause mortality risk excluding deaths occurring the first 24 months of follow up. Imputed data^c^ (*n* = 198,383; 4714 deaths)

	With 1 hr of:							
1. Isotemporal Substitution Model-*Replace 1 hr of*:	Sleeping (<=7 hrs)	Sleeping (>7 hrs)	Screen-time	Sitting	Standing	Walking	MVPA	Total activity
A. Sleeping (<=7 hrs)	-	-	1.02 (0.97–1.07)	1.03 (0.98–1.08)	0.99 (0.94–1.03)	0.89 (0.78–1.01)	0.90 (0.83–0.98)	1.00 (0.97–1.04)
B. Sleeping (>7 hrs)	-	-	0.96 (0.94–0.99)	0.97 (0.94–0.99)	0.93 (0.91–0.96)	0.80 (0.73–0.87)	0.89 (0.85–0.93)	1.04 (1.02–1.07)
C. Screen-time	0.95 (0.90–1.00)	1.05 (1.02–1.08)	-	1.01 (0.99–1.03)	0.97 (0.95–0.98)	0.84 (0.79–0.90)	0.91 (0.88–0.95)	1.01 (1.00–1.03)
D. Sitting	0.94 (0.90–0.99)	1.04 (1.01–1.06)	0.99 (0.97–1.01)	-	0.96 (0.95–0.97)	0.84 (0.78–0.9)	0.90 (0.87–0.94)	1.02 (1.01–1.03)
E. Standing	0.98 (0.94–1.04)	1.08 (1.05–1.11)	1.03 (1.02–1.05)	1.04 (1.03–1.06)	-	0.87 (0.81–0.94)	0.94 (0.91–0.98)	0.98 (0.97–0.99)
F. Walking	1.13 (1.04–1.23)	1.15 (1.1–1.21)	1.19 (1.11–1.27)	1.20 (1.12–1.28)	1.15 (1.07–1.23)	-	1.08 (1.00–1.17)	0.85 (0.8–0.92)
G. MVPA	1.05 (0.98–1.11)	1.14 (1.09–1.18)	1.10 (1.06–1.14)	1.11 (1.07–1.15)	1.06 (1.02–1.10)	0.93 (0.85–1.00)	-	0.92 (0.89–0.96)
2. Partition model^a^	1.00 (0.97–1.04)	1.06 (1.03–1.09)	1.02 (1.01–1.03)	1.03 (1.02–1.04)	0.97 (0.96–0.98)	0.82 (0.77–0.88)	0.90 (0.87–0.93)	

^a^Adjusted for sex, age, educational level, marital status, urban or rural residence, BMI, smoking status, self-rated health, receiving help with daily tasks for a long-term illness or disability, prevalent disease at baseline (cardiovascular disease, diabetes, or cancer), psychological distress, and mutually adjusted for all activity classes

^b^Adjusted for sex, age, educational level, marital status, urban or rural residence, BMI, smoking status, self-rated health, receiving help with daily tasks for a long-term illness or disability, prevalent disease at baseline (cardiovascular disease, diabetes, or cancer), psychological distress, mutually adjusted for all activity classes, and total time in all activity classes

^c^Multiple imputation to replace missing time of the activity classes (based on age, sex, and non-missing other activity classes variables)

## Discussion

This is the first large-scale epidemiological study examining the replacement effects of sedentary time and other time-dependent behaviours on all-cause mortality using statistical modelling. Both screen time and total sitting time were independently associated with increased mortality risk, while standing, walking, and MVPA were associated with decreased mortality risk. We found beneficial associations for replacing sedentary time with equal amounts of sleeping (in participants who sleep < 7 h/day), standing, walking, and MVPA. Our results were robust to multiple measures that we took to minimize the chances of reverse causality.

Public health physical activity recommendations [21] are largely based on epidemiological evidence of non-substitution models. Our analyses suggest that the ISM [10] offers richer and more specific information than previous “static” methods. Because there seems to be a variation in the associations of most behaviours with mortality depending on the displaced activity, existing epidemiological evidence may underestimate the benefits of physical activity and the harms of sedentary behaviour. For example, the partition models showed that each hour/day of sitting is linked to an increased mortality risk of 3 % (2–4 %), but once the displaced activity is taken into account, this increased to 5 % (4–6 %) and 17 % (11–23 %) for displacing equal amounts of standing and MVPA, respectively.

For interventional targets, the most common scenario is that programs seek to reduce sitting and screen time and promote MVPA [22]. Interventions aimed at specifically replacing sitting with standing are less common and are mostly restricted to the occupational office-based environment [23]. Our results suggest that standing time also may be an additional promising interventional target. Given the evident difficulties in promoting MVPA at a population level, this approach might be promising for certain situations and populations/clinical groups where physical activity messages are difficult to disseminate. The only epidemiological study, to our knowledge, that specifically examined the associations of standing time with mortality [24] used a non-substitutional approach and found that the proportion of daily time spent on standing is associated with all-cause and CVD mortality in nearly inverse dose–response manner among the physically inactive participants only [24]. Our partition models suggested an independent beneficial effect of standing on mortality (3 % decrease in risk per hour of standing in the whole sample), and this association was present in both those who met and did not meet the physical activity recommendations. These beneficial associations of standing with mortality were even more substantial when standing time displaced SB. The cardiometabolic properties of standing have not been studied extensively, perhaps due to the absence of an established mechanism through which it may benefit health. A rodent model-based hypothesis put forward over a decade ago suggested that prolonged sitting causes dramatic reductions of lipoprotein lipase activity compared to standing up or ambulating [25], although human studies that manipulated experimentally sitting refute this hypothesis as there appears to be no benefit from replacing sitting with standing on blood lipid variables [26–28]. Instead, replacing sitting with standing [26, 29] or light-intensity walking [30] may improve postprandial blood glucose responses and energy expenditure [29].

Assuming that our findings represent causal effects, substituting sitting with standing or other light-intensity PA  may have considerable public health and clinical care implications, e.g. replacing three hours of sitting per day with standing may be associated with a cumulative decrease of 12–18 % in all-cause mortality risk. Working hours account for over half of total waking time [31] and workers in many professions spend on average more than 70 % of their work time sitting [32]. Unlike promoting physical activity, substitution of desk-based sitting for standing is a relatively straightforward intervention that has no additional time and location requirements. Objective British [33] and US data [34] indicate that on average people aged 70 years and older spent approximately 65–80 % of their waking time being sedentary. Substituting sedentary time with standing and light-intensity activity in this challenging age group may be promising.

The strengths of our study are the large population-based sample, the availability of data on a broad range of PA-related behaviours and sleep that collectively account for the majority of the 24-hour daily cycle, the novel statistical approach that allows examination of replacement effects, and the multiple measures we took to reduce the chances of reverse causation and confounding. While the relatively low 45 and Up response rate (19 % [13]) may be seen as a threat to the generalizability of our findings, it is unlikely that our results were materially compromised as relative risks based on internal comparisons are not dependent on the representativeness of the cohort. A previous analysis that compared a broad range of exposure-outcome associations in the 45 and Up cohort with another New South Wales population study with much higher response rate (~60 %) found that the relative risk estimates in the two studies were almost identical in both magnitude and direction [35]. A limitation of our work was that the exposures were only measured at baseline and our data did not reflect complete time-use as we lacked information on other light-intensity physical activity (non-walking/non-standing activities <1 · 5 MET). Our measure of screen time did not differentiate between recreational and occupational screen time, as the health effects of TV and other recreational screen [5, 6] may be different to those of e.g. occupational computer use. Our study is based on statistical modeling and not on actual replacements of one activity with another. Our time-dependent variables were all self-reported although many have been previously validated and our results further support their convergent validity. Standing, sitting, and sleeping were measured on a different scale to walking and MVPA and this may have affected comparability of the responses to some extent. Physical activity may have been over-reported and sitting time under-reported due to social desirability bias. There was no information on walking pace so it is not possible to make inferences about its intensity.

## Conclusions

Sedentary time was associated with increased risk and physical activity with decreased risk for all-cause mortality in adults aged ≥45 years. The magnitude of these effects varied broadly according to the behaviour displaced and there was evidence of an activity intensity-graded response. Isotemporal substitution modelling offers richer and more specific insights into the associations of each time-dependent activity class with mortality compared to traditional non-substitutional approaches.

## Additional file

Additional file 1:
**Contents: Supplementary Methods; Figure S1; Figure S2; Table S1; Table S2; Table S3; Table S4; Table S5; Table S6; Table S7;Table S8;Table S9; Table S10. ** (DOCX 230 kb)

## Abbreviations

HR: Hazard ratio
ISM: Isotemporal substitution model
MVPA: Moderate to vigorous physical activity
RBDM: Registry of Births, Deaths, and Marriages
SB: Sedentary behaviour
