# Supplementary material for: All-cause mortality effects of replacing sedentary time with physical activity and sleeping using an isotemporal substitution model: a prospective study of 201,129 mid-aged and older adults
Source: Int J Behav Nutr Phys Act. 2015 Sep 30;12:121. doi: 10.1186/s12966-015-0280-7 (PMC4589071; doi:10.1186/s12966-015-0280-7)
Supplement: Additional file 1: — Contents: Supplementary Methods; Figure S1; Figure S2; Table S1; Table S2; Table S3; Table S4; Table S5; Table S6; Table S7;Table S8;Table S9; Table S10. (DOCX 230 kb) [file 12966_2015_280_MOESM1_ESM.docx]

**ADDITIONAL FILE 1 – Supplemental Material**

**Supplementary Methods: Unabridged description of data cleaning, handling, variable derivation and statistical testing procedures**

*Deriving and cleaning time-dependent activity class variables*

Time use in the 45 and Up baseline questionnaire was derived from two separate questions. The first item participants to detail the number of hours per day spent sitting, standing, sleeping, and spent in front of a computer screen or TV. The second question asked participants for the time per week (and sessions) of walking, moderate physical activity, and vigorous activity.

The time spent in different forms of physical activity was processed to minimize missing values[^1^](#_ENREF_1), and then each weekly total was divided by 7 to derive a daily estimate of the average amount of time spent in each activity. As there may have been variation in the way participants responded to the other time use question, so we undertook a series of checking and cleaning steps to harmonize the data:

1. If participants had answered all time-use questions but screen-time, we imputed 0 into the hours screen-time per day.
2. If the total of sitting, standing, and sleeping was between 23 and 25 hours, we assumed that they had entered amount of screen-time separately to other activities (i.e. as a sub-component of sitting time), and separated sitting time into non-screen sitting time and screen sitting time.
3. If time spent per day in physical activity (total) was less than reported standing time, we assumed that they had included physical activity as part of their standing time, and so we separated this from their reported standing time (modified standing time = original hours reported standing – total physical activity hours).

After these harmonization steps we then excluded any data where all original activity class variables (sitting, screen-time, standing, sleeping, walking , moderate-to-vigorous physical activity) were missing. We also excluded any participants who reported a total time-use of less than 20 hours, or more than 28 hours. Table S1 below shows the mean, standard deviations and median values for each harmonized time use variable.

Preliminary analyses confirmed a ‘u-shaped’ relationship between sleeping duration and risk of death, with higher risk in participants with low levels of sleeping (i.e. 6 hours or below) and high amounts of sleeping (10 hours plus). The isotemporal model depends on a linear association of the time variable with the outcome, so we created a piecewise sleeping variable that separately estimated the effect of sleeping on mortality based on a cut-point. We fitted sleeping duration to mortality (binomial distribution) with a smoothing (2 df)in proc gam (SAS 9.3). We confirmed the u-shaped relationship between sleep duration and mortality for sleep duration between 0 and 14 hours (Supplementary Methods Figure 1). The smoothing spline extends beyond 14 hours but only 0.1% (n=193) had a value greater than this, so this should have negligible influence on the results.

| Table S1: Means, standard deviations, and medians of the harmonised time use variables. | | |
| --- | --- | --- |
| Time use variable | Mean (SD) | Median |
|  | (hours/day) |  |
| Sleeping | 7.7 (1.3) | 8.0 |
| Screen time | 4.2 (2.5) | 4.0 |
| Sitting | 5.0 (2.7) | 5.0 |
| Standing | 4.1 (3.0) | 3.4 |
| Walking | 0.4 (0.5) | 0.24 |
| MVPA | 0.8 (1.0) | 0.44 |
| Total Time | 22.0 (3.8) | 23.0 |

**Supplementary Methods Figure 1**


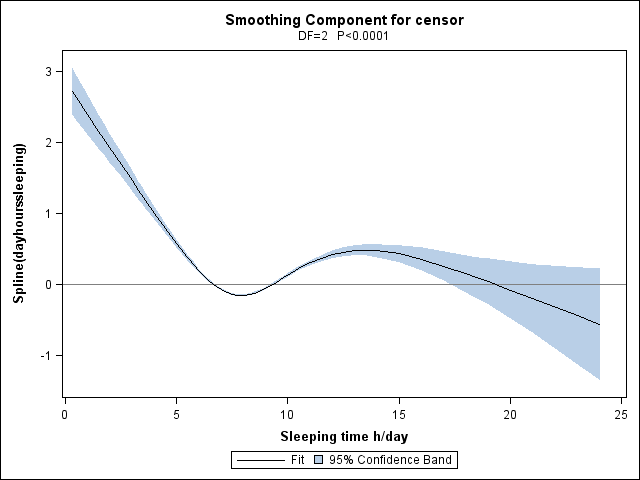


Using 7 hours of sleeping as our cutpoint, we created a piece-wise variables to be able to estimate the additional and isotemporal effects of sleeping.

For those reporting 7 or less hours of sleep: $\begin{matrix} \text{sleep\_a}=\text{sleep duration} \\ \text{sleep\_b}=0 \end{matrix}$

For those reporting greater than 7 hours of sleep: $\begin{matrix} \text{sleep\_a}=0 \\ \text{sleep\_b}=\text{sleep duration}-7 \end{matrix}$

These variables were both included in the partition model to estimate the additional effect of sleeping duration, and in the isotemporal models where other time-use variables were dropped to estimate the effect of substituting another activity for sleeping. The coefficients b_1_ and b_2_ represent the effect of substituting sleep for the activity dropped from the model (e.g. screen time) in those that have 7 hours of sleep or less, and those with more than 7 hours, respectively.

$$\begin{matrix} \log h_{i}(t)=b_{0}(t)+b_{1}(\text{sleep\_a})+b_{2}(\text{sleep\_b})+b_{3}(\text{sitting})+b_{4}(\text{standing}) \\ +b_{5}(\text{walking})+b_{6}(\text{MVPA})+b_{7}(\text{Total})+(\text{covariates}) \end{matrix}$$

To estimate the effect of substituting sleep for another activity, we created a version of all the other time use variables specific to the amount of sleep. For example, for standing time:

For those reporting 7 or less hours of sleep: $\begin{matrix} \text{stand\_a}=\text{standing time} \\ \text{stand\_b}=0 \end{matrix}$

For those reporting greater than 7 hours of sleep: $\begin{matrix} \text{stand\_a}=0 \\ \text{stand\_b}=\text{standing time} \end{matrix}$

This is the equivalent of an interaction term between standing time and a dichotomous variable with the value of 1 if the participant reported more than 7 hours of sleep; i.e. it will estimate the effect of substation separately depending on the level of sleep. A linear combination of standing time and this interaction term will give the same estimate as creating a second standing time variable for those with greater than 7 hours of sleep.

These were analyzed similarly, to the isotemporal model above, except with the two versions of each of the time-use variables.

This strategy was used to be able to perform the same adjustments for socio-demographic factors in the partition and isotemporal models in each cell of the isotemporal analysis table. Stratifying by sleeping time would allow all of these adjustments to also vary according to sleeping time and confound the results with differences in the adjustments for confounding.

*Data handling including multiple imputation*

As 13053 participants had at least one of the harmonized time-use behavioural variables missing, we used multiple imputation (SAS 9.3, Proc MI) to impute these missing values. The Expectation-Maximisation algorithim was used to estimate initial values for the Markov-chain Monte Carlo procedure to create 30 imputations of the dataset[^2^](#_ENREF_2). Moderate and vigorous PA were combined into MVPA to improve linearity of the association between PA and mortality. We chose to treat walking as a separate variable because a) the walking questions did not specify intensity, and b) the greater specify of the replacement affects we can calculate by entering it as a separate variable to MVPA . Due to the established U-shaped association between sleeping and all-cause mortality[^3^](#_ENREF_3) [^4^](#_ENREF_4) and the ISP requirement for a linear or nearly-linear association between each time-dependent exposures and the outcome, all Cox models described below treated sleeping as a piecewise variable with a breakpoint at 7 hours (≤7 hours/day and > 7 hours/day). The breakpoint was determined by modelling hazard and sleeping time with a generalised additive model, and selecting the point where the slope changed from negative to positive (see appendix A for more details). Each of the piecewise sleeping variables had an approximately linear association with mortality. All exposure variables were converted into hours per day.

*Statistical analyses*

The association between each activity class and risk of death was analyzed using Cox proportional hazards regression models[^5^](#_ENREF_5). The piecewise sleeping variables were entered as two separate variables; one estimated the effect of sleeping in those who had 7 or less hours of sleep, and those who slept more than 7 hours (see Appendix A).

Survival time (in weeks) was measured as the time from baseline to death or the censor point. Death from all causes was the outcome variable, and each activity type was modelled in one-hour intervals. Prior to Cox analyses with the isotemporal model, the following assumptions were tested: i) all potential confounders entered in the models met the proportional hazards assumption; ii) the time-dependent behavioral class variables was linear (tested with addition of power terms); iii) there were no interactions between the activity class variables and the covariables. All of these variables were appropriate for further use in the isotemporal model. Because interactions of sex and age were not statistically significant (all p > 0.10, not shown) results are presented for the entire sample.

*Partition model*

First, we estimated the partition model, which estimates each component of time while keeping others constant:

$$\begin{matrix} \log h_{i}(t)=b_{0}(t)+b_{1}(\text{sleep\_a})+b_{2}(\text{sleep\_b})+b_{3}(\text{sitting})+b_{4}(\text{standing}) \\ +b_{5}(\text{walking})+b_{6}(\text{MVPA})+(\text{covariates}) \end{matrix}$$

The partition model, that is the standard type of Cox analysis in sleep, SB and physical activity research, assumes that each activity class is added rather than substituted with another activity to create a day potentially longer than 24 hours.[^6^](#_ENREF_6)

*The isotemporal substitution model*

The isotemporal substitution model makes the more realistic assumption that an increase in duration of one activity class will be accompanied by a decrease of equal duration (isotemporal) in another activity class while total time is kept constant. [^7^](#_ENREF_7) For example, to estimate the effect of substituting one hour of standing for screen-time, screen-time is removed from a model adjusted for total time as follows:

$$\begin{matrix} \log h_{i}(t)=b_{0}(t)+b_{1}(\text{sleep\_a})+b_{2}(\text{sleep\_b})+b_{3}(\text{sitting})+b_{4}(\text{standing}) \\ +b_{5}(\text{walking})+b_{6}(\text{MVPA})+b_{7}(\text{Total})+(\text{covariates}) \end{matrix}$$

In the above example, the resulting HR for standing will demonstrate whether replacing screen time with standing is beneficially (if HR<1.00) or detrimentally (if HR>1.00) associated with all-cause mortality. For estimating the effect of substituting sleeping time, this was expanded to split each other time variable according to whether the participant was above or below the change-point (see Appendix A for details).

*Sensitivity analyses*

We repeated analyses in the unimputed dataset only on those participants who had data in all variables (n= 188,071; 6,760 events). We also performed a series of further sensitivity analyses to examine the robustness of our results to: a) exclusions of certain sub-groups that may be prone to reverse causation; b) alternative manipulations of the SB variables. All survival analyses were repeated for the subgroups of participants with cardiovascular disease (including heart disease, stroke, and thrombosis), or diabetes, or cancer at baseline. Because of the relatively short follow-up, we repeated analyses after excluding participants who died in the first 24 months of follow-up (n = 4714). To exclude the possibility that participants’ responses assumed screen time as a subcomponent of total sitting time we repeated analyses with screen time omitted from the partition and ISP models. Assuming that screen time and sitting were reported as two completely distinct activity classes, we repeated analyses with a total sedentary behaviour time variable operationalized as the sum of the two. We also performed another two sensitivity analyses where we handled the sleep varaible differently: first, we stratified the main analyses by sleep time level (≤7 hours/day and >7 hours/day); we repeated the main analysis using a different cut-off for the piecewise sleep variable (8 hours/day). Analyses were performed using SAS Software version 9.3 (SAS Institute Inc., Cary, NC). The reporting of this study conforms to the STROBE statement[^8^](#_ENREF_8).

**Figure S1:** flowchart of final study sample derivation


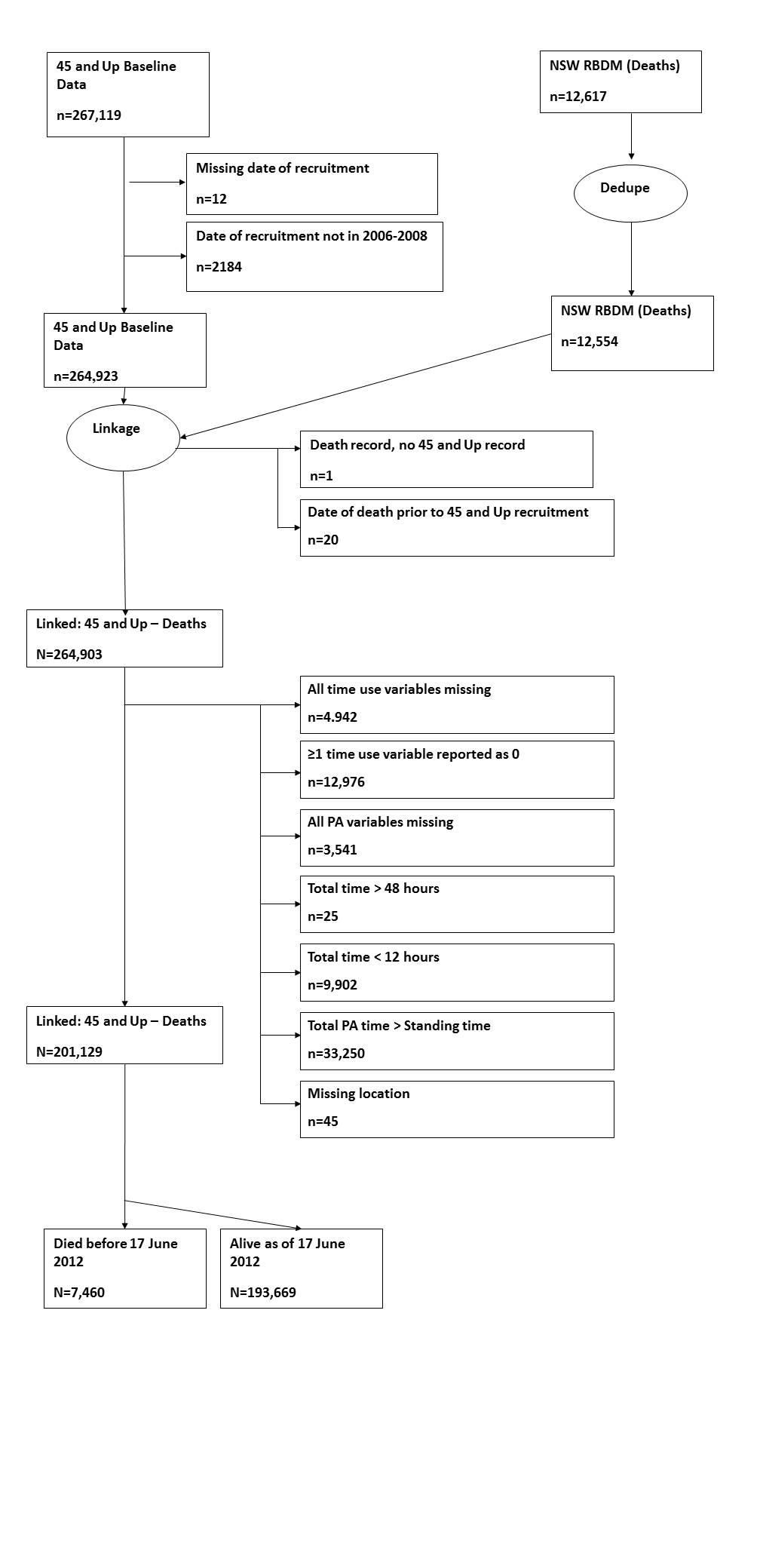


| **Figure S2:** Per-hour Hazard Ratios and 95% Confidence Intervals for all-cause mortality risk^a^. Partition model (A^b^); Isotemporal substitution models for replacing one hour of sleeping for those reporting ≤7 hrs/day of sleep (B^c^), sleeping for those reporting >7 hrs/day of sleep (C^c^), screen time (D^c^), sitting (E^c^), standing (F^c^)walking (G^c^), and MVPA (H^c^) |
| --- |
|  |
|  |
|  |
|  |
|  |
|  |
|  |
|  |
| ^a^ Multiple imputation to replace missing time of the activity classes (based on age, sex, and non-missing other activity classes variables).  ^b^ Adjusted for sex, age, educational level, marital status, urban or rural residence, BMI, smoking status, self-rated health, receiving help with daily tasks for a long-term illness or disability, prevalent disease at baseline (cardiovascular disease, diabetes, or cancer ), psychological distress, and mutually adjusted for all other activity classes.  ^c^ Adjusted for sex, age, educational level, marital status, urban or rural residence, BMI, smoking status, self-rated health, receiving help with daily tasks for a long-term illness or disability, (cardiovascular disease, diabetes, or cancer ), psychological distress, mutually adjusted for all other activity classes, and total time in all activity classes.  **MVPA**: moderate-to-vigorous physical activity |

| **Table S2: Correlation matrix (via z-score correlation with multiple imputation) between different activity classes (n=201,129)** | | | | | | | | |
| --- | --- | --- | --- | --- | --- | --- | --- | --- |
|  | **Sleeping (≤ 7 hrs)** | **Sleeping (>7hrs)** | **Screen-time** | **Sitting** | **Standing** | **Walking** | **MVPA** | **Total time** |
| A. Sleeping (≤ 7 hrs) | 1 | - | 0.065 | 0.098 | 0.027 | 0.006 | -0.008 | -0.098 |
| B. Sleeping (>7hrs) |  | 1 | -0.059 | -0.101 | -0.061 | -0.026 | -0.019 | 0.142 |
| C. Screen-time |  |  | 1 | 0.236 | -0.246 | -0.066 | -0.104 | 0.57 |
| D. Sitting |  |  |  | 1 | -0.416 | -0.106 | -0.163 | 0.445 |
| E. Standing |  |  |  |  | 1 | -0.035 | -0.037 | 0.295 |
| F. Walking |  |  |  |  |  | 1 | 0.173 | 0.037 |
| G. MVPA |  |  |  |  |  |  | 1 | 0.071 |
|  |  |  |  |  |  |  |  | 1 |

| **Table S3. Independent^a^ and isotemporal substitution^b^ effects of sleeping, screen time, sitting, walking, non-walking moderate physical activity, vigorous physical activity on all-cause mortality risk. Unimputed data (N=188,071; 6760 deaths)** | | | | | | | | |
| --- | --- | --- | --- | --- | --- | --- | --- | --- |
|  | **With 1hr of:** | |  |  |  |  |  |  |
| **1. Isotemporal Substitution Model-**  ***Replace 1hr of*:** | **Sleeping (≤ 7 hrs)** | **Sleeping (>7hrs)** | **Screen-time** | **Sitting** | **Standing** | **Walking** | **MVPA** | **Total activity** |
| A. Sleeping (≤7hrs) |  |  | 1.02  (0.98 - 1.06) | 1.03  (0.99 - 1.07) | 0.98  (0.95 - 1.02) | 0.93  (0.84 - 1.03) | 0.91  (0.86 - 0.98) | 1.01  (0.97 - 1.04) |
| B. Sleeping (>7hrs) |  |  | 0.95  (0.93 - 0.97) | 0.96  (0.94 - 0.98) | 0.92  (0.90 - 0.94) | 0.80  (0.74 - 0.86) | 0.84  (0.81 - 0.87) | 1.06  (1.04 - 1.08) |
| C. Screen-time | 0.95  (0.91 - 0.99) | 1.06  (1.04 - 1.09) |  | 1.01  (1.00 - 1.03) | 0.96  (0.95 - 0.98) | 0.86  (0.81 - 0.92) | 0.89  (0.86 - 0.92) | 1.01  (1.00 - 1.02) |
| D. Sitting | 0.94  (0.90 - 0.98) | 1.05  (1.03 - 1.07) | 0.99  (0.97 – 1.00) |  | 0.95  (0.94 - 0.96) | 0.85  (0.80 - 0.90) | 0.88  (0.85 - 0.90) | 1.03  (1.02 - 1.04) |
| E. Standing | 0.98  (0.94 - 1.03) | 1.10  (1.08 - 1.13) | 1.04  (1.02 - 1.05) | 1.05  (1.04 - 1.06) |  | 0.90  (0.85 - 0.95) | 0.92  (0.89 - 0.95) | 0.98  (0.97 - 0.99) |
| F. Walking | 1.10  (1.02 - 1.18) | 1.17  (1.12 - 1.22) | 1.16  (1.09 - 1.23) | 1.17  (1.11 - 1.24) | 1.12  (1.05 - 1.18) |  | 1.03  (0.96 - 1.10) | 0.87  (0.82 - 0.93) |
| G. MVPA | 1.07  (1.01 - 1.12) | 1.18  (1.14 - 1.22) | 1.13  (1.09 - 1.16) | 1.14  (1.11 - 1.18) | 1.09  (1.05 - 1.12) | 0.97  (0.91 - 1.04) |  | 0.90  (0.87 - 0.93) |
| **2.** **Partition model**^†^ | 1.01  (0.97 - 1.04) | 1.08  (1.05 - 1.1) | 1.02  (1.01 - 1.03) | 1.03  (1.03 - 1.04) | 0.97  (0.96 - 0.98) | 0.83  (0.78 - 0.88) | 0.88  (0.85 - 0.9) |  |
| ^a^ Adjusted for sex, age, educational level, marital status, urban or rural residence, BMI, smoking status, self-rated health, receiving help with daily tasks for a  long-term illness or disability, psychological distress, and mutually adjusted for all activity classes  ^b^ Adjusted for sex, age, educational level, marital status, urban or rural residence, BMI, smoking status, self-rated health, receiving help with daily tasks for a  long-term illness or disability, psychological distress, total time in all activity classes, and mutually adjusted for all activity classes. | | | | | | | | |

| **Table S4. Independent^a^ and isotemporal substitution^b^ effects of sleeping, screen time, sitting, walking, and non-walking moderate to vigorous physical activity on all-cause mortality risk. Participants who had cardiovascular disease, or diabetes, or cancer at baseline (Imputed data^c^, n = 57,449; 4770 deaths)** | | | | | | | | |
| --- | --- | --- | --- | --- | --- | --- | --- | --- |
|  | **With 1hr of:** | |  |  |  |  |  |  |
| **1. Isotemporal Substitution Model-**  ***Replace 1hr of*:** | Sleeping (≤ 7 hrs) | Sleeping (>7hrs) | Screen-time | Sitting | Standing | Walking | MVPA | Total activity |
| A. Sleeping (≤ 7 hrs) |  |  | 1.01  (0.96 - 1.06) | 1.02  (0.98 - 1.07) | 0.96  (0.91 - 1.01) | 0.92  (0.80 - 1.04) | 0.87  (0.8 - 0.94) | 1.01  (0.98 - 1.05) |
| B. Sleeping (>7hrs) |  |  | 0.94  (0.92 - 0.97) | 0.95  (0.93 - 0.98) | 0.91  (0.89 - 0.93) | 0.75  (0.68 - 0.82) | 0.81  (0.77 - 0.86) | 1.06  (1.04 - 1.08) |
| C. Screen-time | 0.99  (0.94 - 1.05) | 1.06  (1.04 - 1.09) |  | 1.01  (1 .00- 1.03) | 0.96  (0.95 - 0.98) | 0.83  (0.77 - 0.89) | 0.86  (0.83 - 0.9) | 1.01  (1.00- 1.02) |
| D. Sitting | 0.98  (0.93 - 1.03) | 1.05  (1.02 - 1.07) | 0.99  (0.97 – 1.00) |  | 0.95  (0.94 - 0.96) | 0.82  (0.76 - 0.88) | 0.85  (0.82 - 0.89) | 1.02  (1.01 - 1.03) |
| E. Standing | 1.03  (0.98 - 1.09) | 1.11  (1.08 - 1.14) | 1.04  (1.02 - 1.06) | 1.05  (1.04 - 1.07) |  | 0.86  (0.8 - 0.93) | 0.90  (0.86 - 0.94) | 0.97  (0.96 - 0.98) |
| F. Walking | 1.20  (1.10 - 1.31) | 1.15  (1.1 - 1.21) | 1.21  (1.12 - 1.30) | 1.22  (1.14 - 1.31) | 1.16  (1.08 - 1.25) |  | 1.04  (0.95 - 1.14) | 0.84  (0.78 - 0.9) |
| G. MVPA | 1.15  (1.08 - 1.23) | 1.18  (1.14 - 1.23) | 1.16  (1.11 - 1.21) | 1.17  (1.13 - 1.22) | 1.11  (1.07 - 1.16) | 0.96  (0.88 - 1.05) |  | 0.87  (0.84 - 0.91) |
| **2.** **Partition model**^a^ | 0.99  (0.98 – 1.00) | 1.08  (1.05 - 1.10) | 1.02  (1.00 - 1.03) | 1.03  (1.02 - 1.04) | 0.96  (0.95 - 0.97) | 0.79  (0.73 - 0.85) | 0.85  (0.81 - 0.88) |  |
| ^a^ Adjusted for sex, age, educational level, marital status, urban or rural residence, BMI, smoking status, self-rated health, receiving help with daily tasks for a  long-term illness or disability, psychological distress, and mutually adjusted for all activity classes  ^b^ Adjusted for sex, age, educational level, marital status, urban or rural residence, BMI, smoking status, self-rated health, receiving help with daily tasks for a  long-term illness or disability, psychological distress, total time in all activity classes, and mutually adjusted for all activity classes.  ^c^ Multiple imputation to replace missing time of the activity classes (based on age, sex, and non-missing other activity classes variables) | | | | | | | | |

| **Table S5. Independent^a^ and isotemporal substitution^b^ effects of sleeping, screen time, sitting, walking, non-walking moderate physical activity, vigorous physical activity on all-cause mortality risk. Excluding deaths occurring the first 24 months of follow up and participants who had diagnosed cardiovascular disease, or diabetes, or cancer at baseline. Imputed data^c^(n=142,768; 1,778 deaths)** | | | | | | | | |
| --- | --- | --- | --- | --- | --- | --- | --- | --- |
|  | **With 1hr of:** | |  |  |  |  |  |  |
| **1. Isotemporal Substitution Model-**  ***Replace 1hr of*:** | Sleeping (≤ 7hrs) | Sleeping (>7hrs) | Screen-time | Sitting | Standing | Walking | MVPA | Total activity |
| A. Sleeping (≤7hrs) |  |  | 1.04  (0.94 - 1.15) | 1.05  (0.95 - 1.16) | 1.01  (0.91 - 1.11) | 0.91  (0.83 - 0.99) | 0.93  (0.84 - 1.02) | 1.00  (0.93 - 1.08) |
| B. Sleeping (>7hrs) |  |  | 0.97  (0.92 - 1.02) | 0.94  (0.9 - 0.99) | 0.91  (0.87 - 0.96) | 0.83  (0.72 - 0.95) | 0.91  (0.84 - 0.98) | 1.07  (1.02 - 1.12) |
| C. Screen-time | 0.87  (0.80 - 0.95) | 1.04  (0.99 - 1.09) |  | 0.99  (0.96 - 1.01) | 0.95  (0.93 - 0.98) | 0.86  (0.77 - 0.96) | 0.93  (0.88 - 0.98) | 1.03  (1.01 - 1.05) |
| D. Sitting | 0.88  (0.81 - 0.96) | 1.05  (1.00 - 1.10) | 1.01  (0.99 - 1.04) |  | 0.97  (0.94 - 0.99) | 0.87  (0.79 - 0.97) | 0.94  (0.89 - 0.99) | 1.02  (1.00 - 1.04) |
| E. Standing | 0.91  (0.84 – 1.00) | 1.09  (1.04 - 1.14) | 1.05  (1.02 - 1.08) | 1.04  (1.01 - 1.06) |  | 0.91  (0.81 - 1.01) | 0.97  (0.92 - 1.03) | 0.98  (0.96 – 1.00) |
| F. Walking | 1.11  (0.98 - 1.26) | 1.20  (1.07 - 1.35) | 1.16  (1.04 - 1.29) | 1.14  (1.03 - 1.27) | 1.10  (0.99 - 1.23) |  | 1.07  (0.95 - 1.22) | 0.89  (0.80 - 0.99) |
| G. MVPA | 1.08  (1.01 - 1.15) | 1.12  (1.05 - 1.2) | 1.08  (1.02 - 1.14) | 1.10  (1.05 - 1.15) | 1.09  (1.05 - 1.13) | 0.93  (0.82 - 1.05) |  | 0.96  (0.91 - 1.01) |
| **2.** **Partition model**^†^ | 1.07  (1.00 - 1.14) | 1.07  (1.02 - 1.12) | 1.03  (1.01 - 1.05) | 1.02  (1.00 - 1.04) | 0.98  (0.96 – 1.00) | 0.89  (0.80 - 0.99) | 0.86  (0.81 - 0.91) |  |
| ^a^ Adjusted for sex, age, educational level, marital status, urban or rural residence, BMI, smoking status, self-rated health, receiving help with daily tasks for a  long-term illness or disability, psychological distress, and mutually adjusted for all activity classes  ^b^ Adjusted for sex, age, educational level, marital status, urban or rural residence, BMI, smoking status, self-rated health, receiving help with daily tasks for a  long-term illness or disability, psychological distress, total time in all activity classes, and mutually adjusted for all activity classes.  ^c^ Multiple imputation to replace missing time of the activity classes (based on age, sex, and non-missing other activity classes variables) | | | | | | | | |

| **Table S6. Independent^a^ and isotemporal substitution^b^ effects of sleeping sitting, walking, and non-walking moderate to vigorous physical activity on all-cause mortality risk. Screen time is assumed to be a component of total sitting and therefore excluded from these models. Imputed data^c^ (n=201,129; 7460 deaths )** | | | | | | | | |
| --- | --- | --- | --- | --- | --- | --- | --- | --- |
|  | **With 1hr of:** | |  |  |  |  |  |  |
| **1. Isotemporal Substitution Model-**  ***Replace 1hr of*:** | Sleeping (≤ 7 hrs) | Sleeping (>7hrs) | Sitting | Standing | Walking | MVPA | Total activity |  |
| A. Sleeping (≤ 7 hrs) | - | - | 1.03  (0.98 - 1.08) | 0.98  (0.94 - 1.03) | 0.89  (0.78 - 1.01) | 0.89  (0.83 - 0.97) | 1.01  (0.97 - 1.04) |  |
| B. Sleeping (>7hrs) | - | - | 0.97  (0.94 - 0.99) | 0.93  (0.91 - 0.96) | 0.79  (0.72 - 0.87) | 0.89  (0.85 - 0.94) | 1.04  (1.02 - 1.07) |  |
| D. Sitting | 0.94  (0.90 - 0.99) | 1.05  (1.03 - 1.07) | - | 0.96  (0.95 - 0.97) | 0.83  (0.77 - 0.89) | 0.91  (0.87 - 0.94) | 1.02  (1.01 - 1.03) |  |
| E. Standing | 0.98  (0.93 - 1.04) | 1.1  (1.08 - 1.13) | 1.04  (1.03 - 1.06) | - | 0.87  (0.81 - 0.93) | 0.94  (0.91 - 0.98) | 0.98  (0.97 - 0.99) |  |
| F. Walking | 1.13  (1.04 - 1.24) | 1.17  (1.13 - 1.22) | 1.20  (1.12 - 1.29) | 1.15  (1.07 - 1.24) | - | 1.09  (1.00 - 1.18) | 0.85  (0.79 - 0.91) |  |
| G. MVPA | 1.04  (0.98 - 1.11) | 1.19  (1.15 - 1.22) | 1.10  (1.07 - 1.15) | 1.06  (1.02 - 1.10) | 0.92  (0.84 – 1.00) | - | 0.92  (0.89 - 0.96) |  |
| **2.** **Partition model**^a^ | 1.01  (0.97 - 1.04) | 1.08  (1.06 - 1.1) | 1.03  (1.02 - 1.04) | 0.97  (0.96 - 0.98) | 0.84  (0.79 - 0.88) | 0.87 (0.85 - 0.9) |  |  |
| ^a^Adjusted for sex, age, educational level, marital status, urban or rural residence, BMI, smoking status, self-rated health, receiving help with daily tasks for a  long-term illness or disability, prevalent disease at baseline ( cardiovascular disease, diabetes, or cancer ), psychological distress, and mutually adjusted for all activity classes  ^b^Adjusted for sex, age, educational level, marital status, urban or rural residence, BMI, smoking status, self-rated health, receiving help with daily tasks for a  long-term illness or disability, prevalent disease at baseline ( cardiovascular disease, diabetes, or cancer ), psychological distress, mutually adjusted for all activity classes, and total time in all activity classes.  ^c^Multiple imputation to replace missing time of the activity classes (based on age, sex, and non-missing other activity classes variables) | | | | | | | | |

| **Table S7. Independent^a^ and isotemporal substitution^b^ effects of sleeping, total sedentary behaviour time, walking, non-walking moderate physical activity, vigorous physical activity on all-cause mortality risk. Sedentary behaviour time calculated as the sum of screen time and sitting. Imputed data^c^ (n=201,129; 7460 deaths )** | | | | | | | |
| --- | --- | --- | --- | --- | --- | --- | --- |
|  | **With 1hr of:** | | | | | | |
| **1. Isotemporal Substitution Model-**  ***Replace 1hr of*:** | Sleeping (≤ 7 hrs) | Sleeping (>7hrs) | Sedentary time |  | Walking | MVPA | Total activity |
| A. Sleeping (≤ 7 hrs) | - | - | 1.02  (0.99 - 1.06) | 0.98  (0.94 - 1.02) | 0.93  (0.84 - 1.03) | 0.90  (0.84 - 0.96) | 1.01  (0.98 - 1.04) |
| B. Sleeping (>7hrs) | - | - | 0.96 (0.94 - 0.98) | 0.92  (0.90 - 0.94) | 0.80  (0.75 - 0.86) | 0.84  (0.81 - 0.87) | 1.06  (1.04 - 1.07) |
| C. Sedentary time | 0.95  (0.91 - 0.98) | 1.05  (1.03 - 1.07) | - | 0.96  (0.95 - 0.97) | 0.86  (0.81 - 0.91) | 0.88  (0.85 - 0.91) | 1.02  (1.01 - 1.02) |
| D. Standing | 0.99  (0.95 - 1.03) | 1.1  (1.08 - 1.13) | 1.04  (1.03 - 1.05) | - | 0.90  (0.85 - 0.95) | 0.92  (0.89 - 0.95) | 0.98  (0.97 - 0.98) |
| E. Walking | 1.10  (1.03 - 1.18) | 1.16  (1.12 - 1.21) | 1.16  (1.10 - 1.23) | 1.11  (1.05 - 1.18) | - | 1.02  (0.96 - 1.09) | 0.88  (0.83 - 0.92) |
| F. MVPA | 1.07  (1.02 - 1.13) | 1.18  (1.14 - 1.22) | 1.14  (1.10 - 1.17) | 1.09  (1.06 - 1.12) | 0.98  (0.92 - 1.04) | - | 0.90  (0.87 - 0.92) |
| **2.** **Partition model**^a^ | 1.01  (0.98 - 1.04) | 1.08  (1.06 - 1.1) | 1.02  (1.02 - 1.03) | 0.97  (0.96 - 0.98) | 0.83  (0.79 - 0.88) | 0.87  (0.85 - 0.9) |  |
| ^a^ Adjusted for sex, age, educational level, marital status, urban or rural residence, BMI, smoking status, self-rated health, receiving help with daily tasks for a  long-term illness or disability, prevalent disease at baseline ( cardiovascular disease, diabetes, or cancer ), psychological distress, and mutually adjusted for all activity classes  ^b^ Adjusted for sex, age, educational level, marital status, urban or rural residence, BMI, smoking status, self-rated health, receiving help with daily tasks for a  long-term illness or disability, prevalent disease at baseline ( cardiovascular disease, diabetes, or cancer ), psychological distress, mutually adjusted for all activity classes, and total time in all activity classes.  ^c^ Multiple imputation to replace missing time of the activity classes (based on age, sex, and non-missing other activity classes variables) | | | | | | | |

| **Table S8. Independent^a^ and isotemporal substitution^b^ effects of sleeping, screen time, sitting, walking, non-walking moderate to vigorous physical activity, on all-cause mortality risk in persons with 7 hours of sleep or less per night on all-cause mortality risk. Imputed data^c^ (n=78132; 2,193 deaths)** | | | | | | | |
| --- | --- | --- | --- | --- | --- | --- | --- |
|  | **With 1hr of:** | | | | | | |
| **1. Isotemporal Substitution Model-**  ***Replace 1hr of*:** | Sleeping (≤7 hrs) | Screen time | Sitting | Standing | Walking | MVPA | Total activity |
| A. Sleeping (≤ 7 hrs) |  | 1.02  (0.98 - 1.05) | 1.04  (1.00 - 1.08) | 1.03  (0.98 - 1.08) | 0.95  (0.85 - 1.05) | 0.95  (0.88 - 1.01) | 0.95  (0.91 - 0.99) |
| B. Screen-time | 0.93  (0.89 - 0.98) |  | 1.01  (0.98 - 1.03) | 0.96  (0.94 - 0.99) | 0.92  (0.83 - 1.01) | 0.88  (0.84 - 0.93) | 1.02  (1 - 1.04) |
| C. Sitting | 0.93  (0.89 - 0.97) | 0.99  (0.97 - 1.02) |  | 0.96  (0.94 - 0.97) | 0.91  (0.83 – 1.00) | 0.88  (0.83 - 0.93) | 1.03  (1.01 - 1.04) |
| D. Standing | 0.97  (0.93 - 1.02) | 1.04  (1.02 - 1.06) | 1.05  (1.03 - 1.07) |  | 0.95  (0.87 - 1.04) | 0.92  (0.87 - 0.97) | 0.98  (0.96 - 1) |
| E. Walking | 1.08  (0.97 - 1.19) | 1.09  (0.99 - 1.2) | 1.10  (1.00 - 1.21) | 1.09 (1.00 - 1.18) |  | 0.97  (0.86 - 1.08) | 0.93  (0.85 - 1.02) |
| F. MVPA | 1.06  (0.99 - 1.13) | 1.13  (1.07 - 1.20) | 1.14  (1.08 - 1.2) | 1.09 (1.03 - 1.15) | 1.04  (0.92 - 1.16) |  | 0.90  (0.85 - 0.95) |
| **2.** **Partition model**^a^ | 0.97  (0.93 - 1.02) | 1.02  (1.00 - 1.04) | 1.03  (1.01 - 1.04) | 0.98  (0.96 – 1.00) | 0.93  (0.85 - 1.02) | 0.9  (0.85 - 0.95) |  |
| ^a^ Adjusted for sex, age, educational level, marital status, urban or rural residence, BMI, smoking status, self-rated health, receiving help with daily tasks for a  long-term illness or disability, prevalent disease at baseline ( cardiovascular disease, diabetes, or cancer ), psychological distress, and mutually adjusted for all activity classes  ^b^ Adjusted for sex, age, educational level, marital status, urban or rural residence, BMI, smoking status, self-rated health, receiving help with daily tasks for a  long-term illness or disability, prevalent disease at baseline ( cardiovascular disease, diabetes, or cancer ), psychological distress, mutually adjusted for all activity classes, and total time in all activity classes.  ^c^ Multiple imputation to replace missing time of the activity classes (based on age, sex, and non-missing other activity classes variables) | | | | | | | |

| **Table S9. Independent^a^ and isotemporal substitution^b^ effects of sleeping, screen time, sitting, walking, non-walking moderate to vigorous physical activity, on all-cause mortality risk in persons with more than 7 hours of sleep per night on all-cause mortality risk. Imputed data^c^ (n= 122,997; 5267 deaths)** | | | | | | | |
| --- | --- | --- | --- | --- | --- | --- | --- |
|  | **With 1hr of:** | | | | | | |
| **1. Isotemporal Substitution Model-**  ***Replace 1hr of*:** | Sleeping (>7 hrs) | Screen time | Sitting | Standing | Walking | MVPA | Total activity |
| A. Sleeping (>7 hrs) |  | 1.02  (0.98 - 1.05) | 1.04  (1.00 - 1.08) | 1.03  (0.98 - 1.08) | 0.95  (0.85 - 1.05) | 0.95  (0.88 - 1.01) | 0.95  (0.91 - 0.99) |
| B. Screen-time | 0.93  (0.89 - 0.98) |  | 1.01  (0.98 - 1.03) | 0.96  (0.94 - 0.99) | 0.92  (0.83 - 1.01) | 0.88  (0.84 - 0.93) | 1.02  (1 - 1.04) |
| C. Sitting | 0.93  (0.89 - 0.97) | 0.99  (0.97 - 1.02) |  | 0.96  (0.94 - 0.97) | 0.91  (0.83 – 1.00) | 0.88  (0.83 - 0.93) | 1.03  (1.01 - 1.04) |
| D. Standing | 0.97  (0.93 - 1.02) | 1.04  (1.02 - 1.06) | 1.05  (1.03 - 1.07) |  | 0.95  (0.87 - 1.04) | 0.92  (0.87 - 0.97) | 0.98  (0.96 - 1) |
| E. Walking | 1.08  (0.97 - 1.19) | 1.09  (0.99 - 1.2) | 1.10  (1.00 - 1.21) | 1.09 (1.00 - 1.18) |  | 0.97  (0.86 - 1.08) | 0.93  (0.85 - 1.02) |
| F. MVPA | 1.06  (0.99 - 1.13) | 1.13  (1.07 - 1.20) | 1.14  (1.08 - 1.2) | 1.09 (1.03 - 1.15) | 1.04  (0.92 - 1.16) |  | 0.90  (0.85 - 0.95) |
| **2.** **Partition model**^a^ | 0.97  (0.93 - 1.02) | 1.02  (1.00 - 1.04) | 1.03  (1.01 - 1.04) | 0.98  (0.96 – 1.00) | 0.93  (0.85 - 1.02) | 0.9  (0.85 - 0.95) |  |
| ^a^ Adjusted for sex, age, educational level, marital status, urban or rural residence, BMI, smoking status, self-rated health, receiving help with daily tasks for a  long-term illness or disability, prevalent disease at baseline ( cardiovascular disease, diabetes, or cancer ), psychological distress, and mutually adjusted for all activity classes  ^b^ Adjusted for sex, age, educational level, marital status, urban or rural residence, BMI, smoking status, self-rated health, receiving help with daily tasks for a  long-term illness or disability, prevalent disease at baseline ( cardiovascular disease, diabetes, or cancer ), psychological distress, mutually adjusted for all activity classes, and total time in all activity classes.  ^c^ Multiple imputation to replace missing time of the activity classes (based on age, sex, and non-missing other activity classes variables) | | | | | | | |

| **Table S10. Independent^a^ and isotemporal substitution^b^ effects of sleeping, screen time, sitting, walking, non-walking moderate physical activity, vigorous physical activity on all-cause mortality risk. The piecewise sleeping variable was defined with 8 hours as a cut-off point. Imputed data^c^ (n=201,129; 7,460 deaths)** | | | | | | | | |
| --- | --- | --- | --- | --- | --- | --- | --- | --- |
|  | **With 1hr of:** | |  |  |  |  |  |  |
| **1. Isotemporal Substitution Model-**  ***Replace 1hr of*:** | Sleeping (≤ 8 hrs) | Sleeping (>8hrs) | Screen-time | Sitting | Standing | Walking | MVPA | Total activity |
| A. Sleeping (≤8hrs) |  |  | 1.01  (0.98 - 1.05) | 1.03  (0.99 - 1.07) | 0.98  (0.94 - 1.02) | 0.93  (0.84 - 1.03) | 0.90  (0.85 - 0.96) | 1.01  (0.98 - 1.04) |
| B. Sleeping (>8hrs) |  |  | 0.95 (0.93 - 0.97) | 0.96  (0.94 - 0.98) | 0.92  (0.90 - 0.94) | 0.81  (0.75 - 0.86) | 0.84  (0.81 - 0.87) | 1.06  (1.04 - 1.07) |
| C. Screen-time | 0.95  (0.90 - 0.99) | 1.06  (1.03 - 1.09) |  | 1.01  (1 - 1.02) | 0.96  (0.95 - 0.98) | 0.87  (0.82 - 0.91) | 0.88  (0.86 - 0.91) | 1.01  (1.00 - 1.02) |
| D. Sitting | 0.95  (0.90 - 0.99) | 1.04  (1.01 - 1.07) | 0.99  (0.98 – 1.00) |  | 0.95  (0.94 - 0.96) | 0.86  (0.81 - 0.9) | 0.87  (0.85 - 0.9) | 1.02  (1.01 - 1.03) |
| E. Standing | 0.99  (0.95 - 1.03) | 1.1  (1.07 - 1.13) | 1.03  (1.02 - 1.04) | 1.04  (1.03 - 1.06) |  | 0.89  (0.85 - 0.94) | 0.91  (0.89 - 0.94) | 0.98  (0.97 - 0.99) |
| F. Walking | 1.09  (1.02 - 1.16) | 1.15  (1.11 - 1.20) | 1.14  (1.08 - 1.21) | 1.16  (1.11 - 1.21) | 1.11  (1.05 - 1.19) |  | 0.99  (0.96 – 1.09) | 0.99  (0.97 - 1.01) |
| G. MVPA | 1.08  (1.01 - 1.15) | 1.16  (1.13 - 1.19) | 1.14  (1.09 - 1.18) | 1.14  (1.11 - 1.18) | 1.09  (1.06 - 1.12) | 0.90  (0.84 - 0.95) |  | 0.96  (0.94 - 0.98) |
| **2.** **Partition model**^†^ | 0.99  (0.98 – 1.00) | 1.07  (1.04 - 1.10) | 1.01  (1.00 - 1.02) | 1.02  (1.01 - 1.03) | 0.98  (0.97 - 0.98) | 0.88  (0.83 - 0.92) | 0.89  (0.87 - 0.92) |  |
| ^a^ Adjusted for sex, age, educational level, marital status, urban or rural residence, BMI, smoking status, self-rated health, receiving help with daily tasks for a  long-term illness or disability, psychological distress, and mutually adjusted for all activity classes  ^b^ Adjusted for sex, age, educational level, marital status, urban or rural residence, BMI, smoking status, self-rated health, receiving help with daily tasks for a  long-term illness or disability, psychological distress, total time in all activity classes, and mutually adjusted for all activity classes. | | | | | | | | |

**Supplementary Material REFERENCES**

1. Banks E, Jorm L, Rogers K, Clements M, Bauman A. Screen-time, obesity, ageing and disability: findings from 91 266 participants in the 45 and Up Study. *Public Health Nutrition.* 2011;14(01):34-43.

2. Little R, Rubin D. *Statistical Analysis with Missing Data.* 2nd ed. New York: John Wiley & Sons; 2002.

3. Cappuccio F, D'Elia L, Strazzullo P, Miller M. Sleep Duration and All-Cause Mortality: A Systematic Review and Meta-Analysis of Prospective Studies. . *Sleep.* 2010;33(5):585–592.

4. Magee CA, Holliday EG, Attia J, Kritharides L, Banks E. Investigation of the relationship between sleep duration, all-cause mortality, and preexisting disease. *Sleep medicine.* 2013;14(7):591-596.

5. Cox D R. Regression models and life tables. *Journal of the Royal Statistical Society: Series B.* 1972;34(2):187-220.

6. Mekary RA, Lucas M, Pan A, et al. Isotemporal Substitution Analysis for Physical Activity, Television Watching, and Risk of Depression. *American Journal of Epidemiology.* August 1, 2013 2013;178(3):474-483.

7. Mekary RA, Willett WC, Hu FB, Ding EL. Isotemporal Substitution Paradigm for Physical Activity Epidemiology and Weight Change. *Am J Epidemiol.* August 15, 2009 2009;170(4):519-527.

8. Elm Ev, Altman DG, Egger M, Pocock SJ, Gøtzsche PC, Vandenbroucke JP. Strengthening the reporting of observational studies in epidemiology (STROBE) statement: guidelines for reporting observational studies. *BMJ.* 2007-10-18 23:01:04 2007;335(7624):806-808.
